# Emerging concepts in salivary tumor pathology: lessons learned from overlaps with non-salivary organs

**DOI:** 10.1007/s00428-026-04560-9

**Published:** 2026-05-02

**Authors:** Abbas Agaimy, Stephan Ihrler

**Affiliations:** 1 Institute of Pathology, Erlangen University Hospital, Friedrich Alexander University of Erlangen-Nuremberg, Erlangen, Germany; 2 Comprehensive Cancer Center, European Metropolitan Area Erlangen-Nuremberg (CCC ER-EMN), Erlangen, Germany; 3 DERMPATH Muenchen, Munich, Germany

The nosology of neoplastic diseases has been dynamically evolving over the years. During this process, mostly organ-specific definitional diagnostic features (morphological/ immunophenotypical and/or molecular) are incorporated to define new and emerging entities and categories. Notably, the pathological phenomena among the different body organs and organ systems are well known to have both convergent and divergent principles and concepts. This is of paramount importance to recognize when defining new entities, so that experts in a particular subspecialty are aware whether to think and conceptualize in an organ-specific (organ-restricted) way or to translate principles long-known and well defined in other organs.

Examples of the “pan-organ concept” are reflected in a couple of old and recently established salivary gland entities that were transferred from other organs including salivary duct carcinoma (well known to recapitulate basic histogenetic principles of breast cancer since the original description in 1968 [1]), secretory carcinoma (recognized as salivary counterpart of what has been historically termed “juvenile breast carcinoma” since 1966 and renamed as “secretory carcinoma of the breast” in 1980 [2]), mucinous adenocarcinoma (recapitulating upper and lower digestive tract adenocarcinoma [3]), sclerosing microcystic adenocarcinoma (recapitulating its skin counterpart [4]) and others. While these tumors mostly share with their other organ counterparts both phenotype and genotype (e.g. secretory carcinoma), others share the phenotype, but not the genotype (e.g. mucinous adenocarcinoma). Conversely, several specific salivary tumor types (microsecretory adenocarcinoma) became secondarily well known in the skin after their salivary delineation.

In this issue of Virchows Archiv, a couple of selective original and review articles are included, that impressively highlight these convergent and divergent diagnostic phenomena in salivary versus non-salivary locations.

The manyfold salivary gland and cutaneous adnexal tumors exhibit a confusing mixture of striking similarities and analogies, but also major discrepancies in their clinical features, terminology, (immune)-histology, and molecular pathology [5]. In a 2-part comprehensive review, Ihrler et al. present a comparison of the different aspects of salivary gland and cutaneous adnexal tumors. In the first part (10.1007/s00428-026-04454-w), the authors describe in an initial explanatory part the basic microscopic und functional anatomy of normal salivary and cutaneous adnexal structures, followed by a topographic assessment of practical differential diagnostic difficulties, anatomically highlighted in the overlapping periparotid and perioral areas.

In the second part (10.1007/s00428-026-04397-2), the authors present and compare a further series of histologically analogous / similar tumor entities. Finally, the article highlights valuable observations in a synoptic comparison of general features among the two organs, pointing out absence of tumors with follicular differentiation in salivary glands, while tumors with acinar and oncocytic differentiation are lacking in the skin adnexa. In general, salivary tumors have a higher proportion of malignancy, are on average larger, are frequently more difficult to resect, and, hence, show a higher propensity for recurrence and metastases, on average with a poorer prognosis. Multifocality with link to hereditary tumour syndromes is not infrequent in cutaneous lesions but is very rare in salivary tumors.

An original article by Agaimy et al. (10.1007/s00428-025-04234-y) addressed the ambiguous and controversial topic of non-sebaceous lymphadenoma (NSLA) histogenesis. Comparing their different morphology pattern, immunophenotype and using NGS, the authors showed, that the majority of so-called lymphoepithelial-type NSLA are indeed displaying a thymus-like phenotype as evidenced by uniform expression of squamous cell markers, bcl2, CD5 and CD117, while lacking SOX10 and other salivary gland markers. Origin from preexistent NSLA with salivary-type (ductal or basaloid) epithelial component was postulated, based on histological findings. Moreover, > 50% of cases harbored inactivating *CYLD* mutations adding to the spectrum of *CYLD*-mutated salivary neoplasms (besides membranous basal cell neoplasms). The biology of these tumors (that showed malignant transformation in 2 cases) remains to be further delineated.

In another original study, Agaimy et al. (10.1007/s00428-025-04369-y) delineated for the first time the occurrence of poroid neoplasms in non-skin head and neck sites with predilection for the salivary glands. These tumors, which span the benign and malignant spectrum of eccrine poroma and porocarcinoma, are predominantly malignant (in contrast to the predominantly benign cutaneous counterparts) and are characterized by similar morphology, immunophenotype and genotype, being uniformly driven by *YAP1::NUTM1/MAML2* gene fusions. The study, while defining a novel tumor category, at the same time highlights the promiscuity of *MAML2* fusions in salivary carcinoma and point to the limitation of *MAML2* FISH (being looked at as specific to mucoepidermoid carcinoma).

Last but not least, Skalova et al. (10.1007/s00428-026-04532-z) present a comprehensive review article, summarizing the most important recent developments in salivary gland tumor pathology, following publication of the last WHO classification. The article illuminates both developments in existing, well-established entities (such as new genotypes in pleomorphic adenoma and illustration of carcinosarcomas, identified as sarcomatoid dedifferentiation of different types of carcinoma ex pleomorphic adenoma) as well as emerging tumor entities, that are looked at as good candidates for the next WHO classification. The article encourages more studies on entities with limited data such as palisading adenocarcinoma to enable inclusion in the future WHO classification.

Taken together, these presented review and original articles and the messages drawn from them clearly highlight, that the challenging and controversial issues in the tumor nosology of a specific organ system cannot be fully solved or addressed, using only or restrictively the principles of that specific organ system and that lessons from taxonomy evolution of other organs should continue to be a major inspiring source. At the same time, organ-limited principles should be acknowledged and unjustified or nonsound generalizations be avoided.

We hope that this minireview series will meet the interest of the broad readership of our journal and in particular those with special interest in head and neck and skin pathology.
